# Evaluation of an infectious‑disease response training program for primary care physicians in Korea using Kirkpatrick’s 4 levels and the Context, Input, Process, and Product model: a mixed‑methods study

**DOI:** 10.3352/jeehp.2025.22.40

**Published:** 2025-12-31

**Authors:** Kyung Hee Chun, Jin Seo Lee, Seon Young Jeong, Young Soon Park

**Affiliations:** 1 Department of Medical Education, Konyang University College of Medicine, Daejeon, Korea; 2 Division of Infectious Disease, Department of Internal Medicine, Kangdong Sacred Heart Hospital, Hallym University College of Medicine, Seoul, Korea; 3 Department of Nursing, Konyang University College of Nursing, Daejeon, Korea; 4 Department of Medical Education, Catholic Kwandong University College of Medicine, Gangneung, Korea; Hallym University, Korea

**Keywords:** Communicable diseases, Delivery of health care, Personal satisfaction, Primary care physicians, Psychometrics, Republic of Korea

## Abstract

**Purpose:**

This study systematically evaluated the effectiveness of a training program designed to enhance infectious disease response capabilities among primary care physicians. Using a mixed-methods design, the evaluation applied Kirkpatrick’s 4-level model and the Context, Input, Process, and Product (CIPP) framework to assess program outcomes and identify areas for improvement.

**Methods:**

The study focused on a national infectious disease training program for primary care physicians conducted in 2022 (N=1,718). Quantitative pre- and post-training data were analyzed from 100 randomly selected participants, along with qualitative data obtained through in-depth interviews with 10 participants. Validated assessment tools, developed by psychometricians and content experts, were used to measure satisfaction (Kirkpatrick level 1), learning achievement (level 2), practical application (level 3), and organizational contribution (level 4).

**Results:**

Overall training satisfaction was high (3.96±0.72). Learning achievement (level 2) demonstrated statistically significant improvement from pre-training to post-training assessments (F=12.922, P<0.001). Scores for practical application (level 3; 3.19±0.86) and organizational contribution (level 4; 3.47±0.70) indicated both strong motivation to apply newly acquired knowledge and institutional readiness to implement response strategies.

**Conclusion:**

This study confirmed that the training program effectively enhanced both individual competencies and organizational response capacity across all 4 Kirkpatrick levels. The integrated application of the Kirkpatrick and CIPP models provided a robust framework for evaluating learning transfer and guiding program improvement. These findings highlight the importance of continued investment in diverse training initiatives, systematic evaluation processes, and the dissemination of successful practices to the broader healthcare community.

## Graphical abstract

[Fig f2-jeehp-22-40]

## Introduction

### Background/rationale

The coronavirus disease 2019 (COVID-19) pandemic demonstrated that the response competency of healthcare professionals is a critical determinant of public health outcomes [1]. Although this recognition led to the introduction of new national-level training programs in Korea, substantial gaps persist across healthcare settings, and the overall support infrastructure remains limited [1,2]. The urgency of strengthening response capacity underscores the need for systematic and multidimensional evaluations to verify training effectiveness and inform continuous program improvement. Kirkpatrick’s 4-level model is a widely used evaluation framework that enables assessment of training transfer from learner reactions (Level 1) and learning outcomes (Level 2) to changes in workplace behavior (Level 3) and organizational results (Level 4) [3]. This model can be complemented by Stufflebeam’s Context, Input, Process, and Product (CIPP) model, a decision-oriented framework designed to assess program relevance, implementation quality, and overall effectiveness [4]. Despite the availability of these comprehensive models, prior evaluations have largely been limited to Level 1 (satisfaction) or Level 2 (learning achievement) outcomes [5-8]. Empirical evidence addressing Level 3 (behavioral change) and Level 4 (organizational impact) remains scarce, largely due to methodological and measurement challenges [9]. This limitation has hindered efforts to establish the true effectiveness of training programs.

### Objectives

Accordingly, this study aimed to systematically evaluate the 2022 Korea Disease Control and Prevention Agency (KDCA)–supported training program for primary care physicians, focusing on infectious disease prevention and management. We applied a mixed-methods design using both Kirkpatrick’s 4-level model and the CIPP model to assess outcomes (including the often-overlooked Levels 3 and 4) and derive quality management strategies. This study was guided by the following research questions (RQs), aligned with Kirkpatrick’s 4 levels of evaluation:

(1) RQ1 (Level 1: reaction): What was the level of participant satisfaction with the infectious disease response training program?

(2) RQ2 (Level 2: learning): What was the impact of the training program on participants’ learning achievement?

(3) RQ3 (Level 3: behavior): What was the impact of the training program on participants’ practical application of learned content in their clinical practice?

(4) RQ4 (Level 4: results): What was the impact of the training program on organizational contributions?

## Methods

### Ethics statement

This study was conducted in strict adherence to research ethics and received approval from the Institutional Review Board (IRB) of Konyang University Hospital (IRB number: KYUH 2021-10-008-004). Written informed consent was obtained from all participants prior to study participation.

### Study design

This study employed a mixed-methods design. A before-and-after study was conducted and reported in accordance with the TREND (Transparent Reporting of Evaluations with Nonrandomized Designs) statement, available at: https://www.cdc.gov/hivpartners/php/trend-statement/index.html. The quantitative component adopted Kirkpatrick’s 4-level model as the primary evaluative framework (Levels 1–4). Level 1 (reaction) evaluation assessed participants’ satisfaction with the training program, with subdomains constructed by integrating Stufflebeam’s CIPP model (Table 1). To assess the effectiveness of the training program at Level 2 (learning), Level 3 (behavior), and Level 4 (results), a pre- and post-test design with a 1-month follow-up assessment was implemented.

### Setting

The training program was conducted from September 26 to October 26, 2022. Quantitative data corresponding to Levels 1–4 were collected using online self-report questionnaires at 3 time points: before the training (September–October 2022), immediately after the training (September–October 2022), and 1 month after the training (October–November 2022). Qualitative data were collected through individual Zoom interviews with high-achieving participants 1 month after the training, between November and December 2022.

### Interventions

The intervention consisted of a 2-hour online continuing medical education (CME) program developed by 4 infectious disease specialists. Detailed information regarding the program structure and content is provided in Supplement 1.

### Participants

The study population comprised 1,718 primary care physicians who participated in the 2022 infectious disease response training program. Among them, 864 individuals who provided consent for evaluation constituted the potential sampling frame. From this pool, 100 participants were selected using random sampling. Demographic analysis of the participants is shown in Table 2.

For the qualitative component (n=10), participants were purposively selected. This subsample (mean age, 53 years; 6 males and 4 females) included specialists in internal medicine (n=2), anesthesiology and pain medicine (n=2), family medicine (n=2), urology (n=2), obstetrics and gynecology (n=1), and pediatrics (n=1).

### Variables

The intervention (exposure) was participation in the 2022 KDCA-supported infectious disease response online CME program for primary care physicians. The primary outcome was Kirkpatrick Level 2 (learning). Secondary outcomes included Kirkpatrick Level 1 (reaction), Kirkpatrick Level 3 (behavior), and Kirkpatrick Level 4 (results). Baseline characteristics and potential covariates included participant sex and clinical practice type (Table 2).

### Data sources

Data collection took place in 2022 in accordance with the program schedule (Dataset 1). Quantitative data for Levels 1–4 were collected through online self-report surveys administered at the 3 predefined time points. Qualitative data were collected 1 month after the training through individual Zoom interviews with 10 participants, selected through purposive sampling based on high performance. This criterion was chosen to identify key informants who could provide rich, detailed insights into the successful application of the training and identify best practices, rather than to achieve statistical generalization.

### Measurement

Level 1 (reaction) was assessed using a 21-item Training Satisfaction Survey developed specifically for this study by integrating Stufflebeam’s CIPP model. The model defined 4 subfactors: Context (necessity), Input (logistics), Process (operation and content), and Product (impact and satisfaction). All items were rated on a 5-point Likert scale. The reliability of the scale is presented in Table 3. Level 2 (learning) was assessed using a 15-item Learning Achievement Test comprising true/false and multiple-choice questions, administered at all 3 assessment points. Level 3 (behavior) was assessed at the 1-month follow-up using a 45-item Practical Application Survey (5-point Likert scale), which measured knowledge dissemination at the individual level and the implementation of response measures at the individual and organizational levels. Level 4 (results) was assessed at the 1-month follow-up using a 15-item Organizational Contribution Survey (5-point Likert scale), which evaluated perceived organizational contribution as a proxy indicator of organizational readiness and system enhancement.

All 4 quantitative instruments (Levels 1–4) were developed specifically for this study. Items were drafted based on existing literature ([10] for Level 1) and consultations with 2 psychometrics experts. Content validity was established through a rigorous expert panel review involving 2 education specialists and 2 infection control specialists. Items were finalized only when all 4 experts rated them as appropriate (defined as ≥4 on a 5-point scale). For the qualitative component, a semi-structured interview guide was developed based on the CIPP framework to derive specific recommendations for program improvement. The content validity of the interview guide was reviewed and refined by 2 education experts.

### Bias

Selection bias is possible because participation in the national training program and consent for evaluation were voluntary; therefore, evaluated participants may have differed systematically from nonparticipants (e.g., higher motivation or greater baseline interest in infection control). To reduce additional selection bias within the evaluation, the quantitative analytic sample was randomly selected from the pool of consenting trainees. However, residual self-selection into both the program and the consent process may still limit the generalizability of the findings.

Information (measurement) bias may have affected Kirkpatrick Levels 1, 3, and 4 because these outcomes relied on self-reported Likert-scale responses, which are vulnerable to social desirability and recall effects. To mitigate these concerns, standardized instruments with explicit anchors were used, and outcomes were assessed at prespecified time points (immediately post-training and at the 1-month follow-up) to minimize long recall intervals. For Level 2, the use of an objective knowledge test reduced susceptibility to social desirability bias relative to self-reported outcomes.

For the qualitative component, sampling bias is possible because interviewees were purposively selected as high performers. While this approach increased the depth of insight into successful implementation, it may have underrepresented barriers experienced by average or lower-performing participants. This limitation should be considered when interpreting the transferability of the qualitative themes.

### Study size

The sample size was determined to ensure sufficient statistical power for the analysis while optimizing data collection efficiency. A priori power analysis indicated power exceeding 95% to detect a medium effect size in repeated-measures analysis of variance (ANOVA). The analysis was conducted using G*Power ver. 3.1 (F test, repeated-measures ANOVA: within factors; Heinrich-Heine-Universität Düsseldorf). Given the single-group design, the number of groups was set to 1. For the power calculation, within-subject changes across 3 time points (pre-intervention, immediately post-intervention, and 1-month follow-up) were modeled for the primary outcome, the learning achievement score. Assuming a medium effect size (f=0.25), a significance level (α) of 0.05, statistical power of 0.95, and a correlation among repeated measures of 0.5, the minimum required sample size was n=43. The final analytic sample in this study (n=100) substantially exceeded this requirement.

There was no investigator-controlled assignment to intervention versus comparison conditions. All trainees received the same training program as implemented at the national level.

### Unit of analysis

The unit of analysis for the quantitative component was the individual physician participant. For the qualitative component, the unit of analysis was also the individual interviewee, and themes were derived from interview transcripts using a CIPP-informed analytic framework.

### Qualitative research

In-depth interviews were conducted using a semi-structured interview guide developed based on the CIPP model. This approach was designed to explore participants’ experiences and perceptions that are difficult to capture through structured surveys alone. Through this process, the Process and Product components were examined in greater depth, including learners’ actual experiences, the appropriateness of program operations, and perceived organizational impacts. This qualitative inquiry complemented the quantitative findings and supported the derivation of specific strategies for program improvement. A mixed-methods approach aligned with Kirkpatrick’s model is shown in Table 4.

### Statistical methods

All quantitative data were analyzed using IBM SPSS Statistics ver. 25.0 (IBM Corp.). Descriptive statistics, including frequencies, means, and standard deviations, were calculated. Repeated-measures ANOVA was used to compare mean scores across the 3 assessment time points. Qualitative data were transcribed verbatim and analyzed using a thematic analysis approach, with the CIPP model applied as an a priori analytical framework. To enhance trustworthiness, coding results were cross-checked by 2 education experts through peer debriefing, and key findings were verified through member checking.

## Results

### Participants

**Fig. 1.**
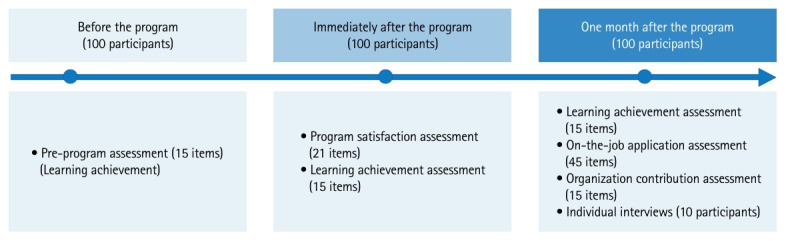
Flow diagram of participant evaluation and interview participation.

Fig. 1 shows the flow diagram of the present study.

### Main results

#### Level 1: Reaction (training satisfaction)

Overall satisfaction with the training program was high (mean=3.96±0.72). As shown in Table 5, participants reported the highest levels of satisfaction for Product items (e.g., willingness to recommend or reattend the program), whereas relatively lower scores were observed for Process items (e.g., program volume and difficulty). Qualitative interviews triangulated these quantitative findings, confirming strong motivation (Context) and high overall satisfaction (Product; mean score 8.5/10). Participants also offered specific and actionable feedback related to Input (e.g., pre-distribution of learning materials) and Process (e.g., the need for adjustable difficulty levels and access to on-demand recordings).

#### Level 2: Learning achievement

As presented in Table 6, repeated-measures ANOVA demonstrated a significant main effect of time on learning achievement (F(2, 198)=12.922, P<0.001, partial ηp^2^=0.115). Post hoc pairwise comparisons using Bonferroni correction indicated that learning achievement scores measured immediately after the intervention and at the 1-month follow-up were significantly higher than pre-intervention scores.

Qualitative interviews further contextualized these results by emphasizing the high relevance and immediate applicability of the content. A key theme was the program’s utility in systematically organizing participants’ existing knowledge. Modules addressing practical prevention strategies (learning outcome [LO]-B) and disease reporting procedures (LO-C) were perceived as particularly useful. Although some participants described the equipment reprocessing module (LO-D) as “dull,” others identified it as new and essential information, underscoring variability in prior knowledge and learning needs (Table 7).

#### Level 3: Practical application (behavior)

Level 3 evaluation, conducted at the 1-month follow-up, yielded a mean score of 3.19±0.86 for individual-level knowledge dissemination (Table 8, Supplement 2). A bimodal response pattern was observed: for common topics (e.g., COVID-19), the most frequent response was “mostly educated” (Level 4), whereas for less common topics (e.g., mpox), the most frequent response was “willing to educate” (Level 3). This pattern suggests a strong motivational effect of the training program. At the individual–organizational level (Table 9, Supplement 3), application of learned content was consistently rated at the “basic” level (Level 3) for both the development (mean=3.13) and implementation (mean=3.13) of response measures across topic categories.

In-depth interviews strongly supported these Level 3 findings, highlighting significant behavioral and attitudinal shifts after the program.

Application of practical knowledge: Participants reported an enhanced state of vigilance. “I learned about the infection control manuals, the proper disinfection procedures, and precautions when treating patients. I now approach (COVID) patients with a heightened sense of awareness.” (A4, A8).

Enhanced patient response competency: The training translated directly into confidence in practice. “Because I learned the knowledge, I can respond to patients with practical confidence and evidence,” and “It was helpful for correcting patients’ misconceptions; I gained confidence in explaining (these topics) to them.” Another noted, “I am using the content for resident and student education.” (A1, A2, A3, A5, A6, A7).

Shift in interprofessional perception: The training also fostered new attitudinal changes. “I used to take it for granted that the nurse cleans up after an endoscopy. After the training on medical equipment reprocessing, I realized how hard they [the nurses] work. I felt a new gratitude for my colleagues and have become more considerate.” (A10).

#### Level 4: Organizational contribution (results)

Level 4 assessment results indicated a positive perceived organizational contribution, with an overall mean score of 3.47±0.70 (Table 10). Participants generally agreed that the program led to tangible increases in awareness and implementation of infection control practices among colleagues and patients, particularly in relation to aerosol prevention (mean=3.60±0.79) and sterilization practices (mean=3.61±0.71).

The interviews provided concrete examples of participants functioning as knowledge brokers and agents of change within their organizations. One participant stated, “I delivered the mpox information to our manager … [and] shared the sterilization knowledge with staff in dentistry and clinical pathology, which helped strengthen our multi-departmental infection control system” (A5).

Another described standardizing departmental practices: “I standardized our department’s infection control practices by creating and distributing a 1-page A4 manual” (A6).

Finally, participants identified a critical future direction for program development: expansion of the training to include nurses and other healthcare staff. This interprofessional approach was viewed as essential for strengthening infection control capacity across the entire organizational system (A7, A8).

## Discussion

### Key results

This mixed-methods study confirmed the effectiveness of the training program across all 4 Kirkpatrick levels. The evaluation demonstrated high satisfaction (Level 1), significant and sustained learning (Level 2), and, most critically, clear evidence of translation into practice (Level 3) and perceived organizational contribution (Level 4). Participants not only acquired knowledge but also reported increased confidence, improved interprofessional appreciation, and active engagement as change agents within their organizations. In addition, the CIPP-based analysis identified actionable areas for improvement, particularly within the Process domain (e.g., program volume and difficulty), thereby demonstrating the added value and synergy of applying both evaluation models concurrently.

### Interpretation

The consistently positive outcomes across all 4 levels suggest that the program’s impact successfully extended from individual learners to the organizational level. The favorable findings at Levels 3 and 4 provide critical evidence that learning outcomes bridged the gap between knowledge acquisition and real-world performance, with meaningful implications for infection control capacity and organizational culture. Furthermore, this study underscores the synergistic value of applying the CIPP model alongside the Kirkpatrick model. While the Kirkpatrick framework confirmed program effectiveness, the CIPP model offered a diagnostic perspective that elucidated how and why the program functioned effectively, yielding specific and actionable strategies for quality management.

However, varying degrees of application were observed across topic areas. The relatively lower scores for mpox-related items in perceived practical application (Level 3) and organizational contribution (Level 4) appear attributable to 2 primary factors. First, at the time of training and analysis, the likelihood of encountering mpox in routine primary care settings was low, resulting in limited perceived relevance. Second, compared with high-frequency and high-risk infectious diseases such as COVID-19, the perceived urgency of responding to mpox was lower. Consequently, the impetus to translate learned content into immediate practice was less pronounced. These findings suggest that the behavioral impact of infectious disease training may vary according to the urgency and contextual relevance of specific topics within real-world clinical environments. Accordingly, when designing CME programs at national or regional levels, careful consideration should be given to aligning educational content and timing with prevailing public health priorities and clinical needs.

### Comparison with previous studies

The findings of this study align with existing literature indicating that infectious disease training enhances not only healthcare professionals’ knowledge and skills but also the transfer of learning into clinical practice [11,12]. Prior studies have further shown that repetitive and participatory training approaches improve knowledge retention and strengthen intentions to implement infection control practices [13,14]. However, a notable limitation of much of the previous research is its emphasis on short-term outcomes, often restricted to Kirkpatrick Levels 1 or 2 [5-8]. A key distinguishing feature of the present study is its longitudinal and multidimensional evaluation. By incorporating a 1-month follow-up assessment and qualitative interviews, this study was able to demonstrate not only sustained learning (Level 2) but also tangible behavior change (Level 3). Moreover, the identification of perceived organizational-level effects (Level 4), including changes in awareness and the adoption of standardized practices, represents an outcome rarely documented in prior studies. These findings suggest that well-designed infectious disease training programs can function as catalysts for improvement, extending beyond individual competency to foster broader enhancements in organizational infection control culture.

### Limitations

This study has several limitations that warrant consideration. First, selection bias arising from voluntary participation may limit generalizability. Although 100 participants were randomly selected from a larger pool of 864 consenting individuals, sampling bias cannot be entirely excluded, and the findings should therefore be interpreted cautiously. Second, the qualitative component relied on purposive sampling of the top 10 performers in the quantitative assessment. While the strong Level 3 and Level 4 outcomes underscore the program’s potential effectiveness, these findings may partly reflect the exceptional baseline characteristics of highly motivated participants. As a result, the experiences and challenges of middle- and lower-performing participants may not have been fully captured, limiting the representativeness of the qualitative insights. Third, reliance on self-reported measures for Levels 3 and 4 introduces the possibility of social desirability bias. In particular, the assessment of Level 4 (results) was based on participants’ perceived organizational impact rather than objective indicators such as return on investment or infection rate metrics. Although collecting such objective data in dispersed primary care settings is inherently challenging, prior research suggests that perceived organizational readiness may serve as a meaningful leading indicator of actual response performance during public health crises [15]. Fourth, the 1-month follow-up period may be insufficient to confirm long-term sustainability of behavioral and organizational change. Longer follow-up intervals of 3 to 6 months or more are generally recommended to assess enduring changes in practice and system-level performance. Finally, the intervention was a web-based program developed by infectious disease specialists and delivered as four 30-minute modules with interactive components and quizzes. Although overall satisfaction was high, feedback indicated a relative need for refinement in program volume, difficulty, and content alignment. To further strengthen field-based infection response competencies, future programs should incorporate diverse educational modalities beyond online delivery, including practice-oriented and problem-solving-focused offline training such as simulations or tabletop exercises.

Notwithstanding these limitations, the mixed-methods design of this study provided a comprehensive and multidimensional assessment of the training program’s effectiveness.

### Suggestion for further studies

Based on these findings, several directions for future research are proposed. First, longitudinal studies are needed to validate the long-term sustainability of learning transfer and behavioral change. Second, future research should prioritize the development of objective assessment tools (e.g., observational checklists or audit-based indicators) to more rigorously evaluate Kirkpatrick Levels 3 and 4. Third, the intervention itself should be further refined through the development of customized modules with adjustable levels of difficulty to better accommodate diverse learner needs. In addition, exploration of hybrid or blended learning models is recommended to enhance learner engagement and facilitate practical application.

The core workforce for infection response is not limited to physicians but includes a broad range of healthcare personnel, such as nurses, administrative staff, and environmental services staff. Accordingly, effective training programs should be expanded to address this wider healthcare workforce. Moreover, given the persistent threat of emerging infectious diseases, effective management increasingly depends on interprofessional collaboration and coordinated response efforts, underscoring the importance of interprofessional education. Ultimately, continued efforts are needed to develop diverse infection response training programs, conduct systematic and theory-informed evaluations, and disseminate successful outcomes to the broader healthcare community.

### Conclusion

This study empirically demonstrated that an infectious disease response training program for primary care physicians can produce substantive outcomes that extend beyond the individual learner. The program not only improved participant satisfaction (Level 1) and learning achievement (Level 2) but also translated into demonstrable behavioral changes in clinical practice (Level 3) and tangible contributions at the organizational level (Level 4). By integratively applying Kirkpatrick’s 4-level model to assess learning transfer and the CIPP model to guide program improvement, this research provided detailed insights into the mechanisms underlying effective learning transfer and identified clear pathways for quality enhancement. This dual-model approach offers a valuable contribution to the advancement of evaluation frameworks in health professions education. Ultimately, the findings of this study illustrate that infectious disease response training is not merely a vehicle for knowledge dissemination but a practical and impactful intervention capable of strengthening both the professionalism of healthcare providers and the overall response capacity of healthcare organizations.

## Figures and Tables

**Figure f2-jeehp-22-40:**
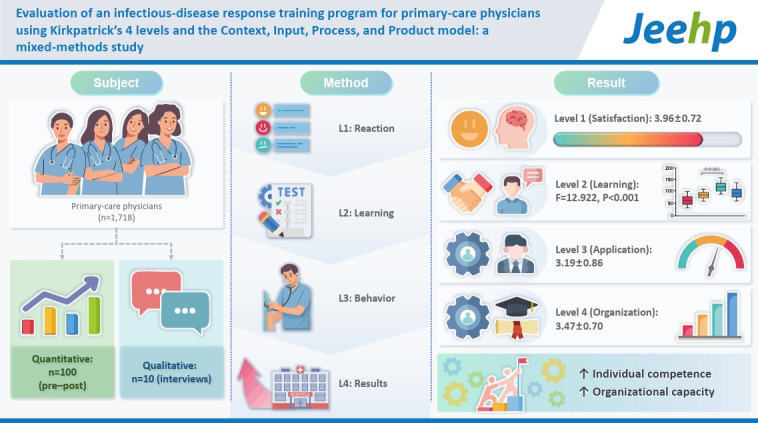


**Table 1.** Research methods and model

CIPP model	Kirkpatrick			
	L1. Program satisfaction survey/interview	L2. Learning achievement assessment	L3. Workplace application assessment	L4. Organizational contribution assessment
Context	●○			
Input	●○			
Process	●○			
Product	●○	●	●	●

●: quantitative method, ○: qualitative method.

**Table 2.** Characteristics of the quantitative sample (n=100) by gender and clinical practice type

Clinical practice type	Male	Female	Total
Endoscopic procedures	11 (11.0)	0 (0.0)	11 (11.0)
Intravenous therapy & injection procedures	12 (12.0)	3 (3.0)	15 (15.0)
Surgery (partial anesthesia)	9 (9.0)	1 (1.0)	10 (10.0)
Surgery (general anesthesia)	10 (10.0)	2 (2.0)	12 (12.0)
Consultation & prescription	39 (39.0)	13 (13.0)	52 (52.0)
Total	81 (81.0)	19 (19.0)	100 (100.0)

Values are presented as number (%).

**Table 3.** Reliability of the training satisfaction survey

Factor (CIPP)	No. of items	Cronbach’s α	Description
Context	3	0.972	Perceptions of program necessity and learning outcomes
Input	3	0.869	Ease of voluntary participation, registration, and guidance
Process	8	0.977	Appropriateness of objectives, content, instructors, platform, Q&A
Product	7	0.987	Impact on personal/organizational competency, overall satisfaction
Total	21	0.989	Reliability for all items

**Table 4.** Overview of quantitative and qualitative evaluation tools

Domain	Instrument	No. of items	Time of administration	Notes
Quantitative (Kirkpatrick)				
Level 1	Training satisfaction (survey)	21	Immediately post-training	5-point Likert scale
Level 2	Learning achievement (test)	15	Pre-training, post-training, 1-month follow-up	True/false multiple-choice items
Level 3	Practical application (survey)	45	1-month follow-up	5-point Likert scale
Level 4	Organizational contribution (survey)	15	1-month follow-up	5-point Likert scale
Qualitative	Semi-structured interview guide	-	1-month follow-up	Individual interviews

**Table 5.** Descriptive statistics for training satisfaction (Level 1) by CIPP domain (n=100)

CIPP domain	Item	Mean±SD
Context	Awareness of program purpose	3.98±0.62
	Perception of program necessity	4.04±0.62
	Awareness of target competency	4.00±0.64
Input	Voluntary participation	3.97±0.78
	Ease of pre-registration	3.98±0.84
	Clarity of pre-program guidance/schedule	3.99±0.70
Process	Clarity of learning objectives	4.00±0.68
	Appropriate program volume/length	3.75±0.77
	Appropriate difficulty level	3.74±0.68
	Content appropriate for achieving goals	3.76±0.77
	Instructor quality	3.99±0.73
	Quality of materials	3.88±0.77
	Online platform stability	4.08±0.75
	Availability of Q&A	3.86±0.79
Product	Helped improve personal competency	4.06±0.69
	Helped improve organizational competency	3.91±0.75
	Helpful for infectious disease response	4.03±0.66
	Motivation to apply learning in organization	3.91±0.74
	Overall satisfaction	4.07±0.77
	Willingness to recommend to colleagues	4.04±0.72
	Willingness to participate in future training	4.09±0.71
Overall		3.96±0.72

SD, standard deviation.

**Table 6.** Learning achievement test scores (Level 2) by time point (n=100)

	Mean±SD	F-value	Post hoc (Bonferroni)
Time point		12.922^***^	2, 3> 1
1. Pre-training	9.53±2.03		
2. Immediate post-training	10.91±2.04		
3. 1-month follow-up	10.48±1.81		

SD, standard deviation.

*** P<0.001.

**Table 7.** Qualitative feedback on learning outcomes (Level 2) from interviews

LOs	Specific content	Participant feedback summary
LO-A (COVID-19)	- Characteristics of new variants	“Helpful for actual patient care.”
	- Vaccine goals	“The module on vaccine goals was most helpful for explaining to patients.”
	- Efficacy of vaccines/prior infection	“Long-COVID patterns were very relevant.”
	- Long COVID patterns	
LO-B (respiratory viruses)	- Main transmission modes	“Helpful for actual practice.”
	- High-risk environments for aerosol spread	“The module on prevention in the clinical environment was the most useful.”
	- Prevention in the clinical setting	
LO-C (emerging diseases & reporting)	- Emergence of novel diseases	“Mpox was interesting, even if rare.”
	- Mpox transmission, PPE	“I had an actual mpox case, so this was extremely helpful.”
	- Mpox clinical presentation, screening	“Learned the correct PPE for suspected cases.”
	- Reportable disease classification and reporting procedures	“The reporting procedures were very useful.”
LO-D (equipment reprocessing)	- Concept and process of reprocessing	“This module felt a bit slow/dull.”
	- Sterilization/disinfection by equipment type	“I've always been interested in sterilization.”
	- Reprocessing workflow in clinics	“This was new information for me; I’m glad I learned it.”
		“For me, LO-D was the most helpful part of the entire program.”

LO, learning outcomes; COVID-19, coronavirus disease 2019; PPE, personal protective equipment.

**Table 8.** Level 3 practical application (knowledge dissemination) by topic category at the 1-month follow-up (n=100)

Topic category	No. of items	Mean±SD
COVID-19 general (1–5)	5	3.33±0.90
COVID-19 prevention (6–8)	3	3.31±0.92
Mpox & PPE (9–10)	2	2.87±1.04
Equipment reprocessing (11–15)	5	3.11±0.95
Overall	15	3.19±0.86

Values are category means calculated from individual item scores. Detailed item-level data and level definitions are provided in Supplement 2. SD, standard deviation; COVID-19, coronavirus disease 2019; PPE, personal protective equipment.

**Table 9.** Level 3 practical application (individual–organizational level) by topic category at the 1-month follow-up (n=100)

Topic category	No. of items	Mean±SD	
		Development	Implementation
COVID-19-related (S1–S4/P1–P4)	8	3.15±0.81	3.15±0.83
Respiratory transmission (S5–S7/P5–P7)	6	3.24±0.87	3.21±0.87
Novel diseases (S8–S10/P8–P10)	6	2.98±0.97	2.93±0.98
Equipment/reporting (S11–S15/P11–P15)	10	3.23±0.85	3.23±0.88
Overall	30	3.13±0.83	3.13±0.85

Values are category means calculated from individual item scores. Detailed item-level data and level definitions are provided in Supplement 3. SD, standard deviation; COVID-19, coronavirus disease 2019.

**Table 10.** Level 4 organizational contribution at the 1-month follow-up: frequencies (n=100, %) and mean

Item (perceived awareness/practice of staff/patients regarding...)	Responses (%)					Mean±SD
	Strongly disagree	Disagree	Neutral	Agree	Strongly agree	
1. Characteristics of new COVID-19 variants	-	11	41	40	8	3.45±0.80
2. Main goals of COVID-19 vaccination	-	12	32	47	9	3.53±0.82
3. Effect of vaccines/prior infection on prevention	-	11	34	45	10	3.54±0.82
4. Patterns of long COVID	-	14	37	42	7	3.42±0.82
5. Main transmission modes of respiratory viruses	1	11	30	46	12	3.57±0.88
6. High-risk environments for aerosol spread	-	8	35	46	11	3.60±0.79
7. Prevention of respiratory virus transmission in clinic	-	7	38	45	10	3.58±0.77
8. Emergence and impact of novel diseases	-	8	43	39	10	3.51±0.78
9. Staff use appropriate PPE for suspected mpox	4	17	38	34	7	3.23±0.95
10. Staff screen/diagnose suspected mpox	4	22	40	26	8	3.12±0.98
11. Staff awareness of reporting classification/methods	1	13	43	35	8	3.36±0.85
12. Staff awareness of reprocessing concept/process	-	6	42	44	8	3.54±0.73
13. Staff select appropriate disinfection/sterilization	-	3	43	44	10	3.61±0.71
14. Staff verify sterilization status of reprocessed equipment	-	5	48	37	10	3.52±0.75
15. Staff apply reprocessing procedures in the institution	-	4	48	39	9	3.53±0.72
Overall						3.47±0.70

SD, standard deviation; COVID-19, coronavirus disease 2019; PPE, personal protective equipment.

## Footnotes

**Data availability**
Data files are available from https://doi.org/10.7910/DVN/VKJTWD Dataset 1. Pre-test and post-test raw scores.

## Supplementary materials

Supplement 1. Detailed information on the infectious disease prevention and management training for primary care physicians.

Supplement 2. Level 3 practical application (individual level: knowledge dissemination) at the 1-month follow-up: frequencies (n=100, %) and mean.

Supplement 3. Level 3 practical application (individual-organizational level) at the 1-month follow-up (n=100).

Supplement 4. Audio recording of the abstract.
