# Supplementary material for: Evaluation of an infectious‑disease response training program for primary care physicians in Korea using Kirkpatrick’s 4 levels and the Context, Input, Process, and Product model: a mixed‑methods study
Source: J Educ Eval Health Prof. 2025 Dec 31;22:40. doi: 10.3352/jeehp.2025.22.40 (PMC13006793; doi:10.3352/jeehp.2025.22.40)
Supplement: Supplementary file 2 — Supplement 1. Detailed information on the infectious disease prevention and management training for primary care physicians. [file jeehp-22-40-suppl1.docx]

**Supplement 1.** Detailed description of the online infectious disease response training program

| Module | Topic | Instructor | Learning objectives | Key contents | Teaching methods |
| --- | --- | --- | --- | --- | --- |
| Module 1 | Epidemiology and recent updates on COVID-19 variants (30 min) | Instructor1 (professor of infectious diseases) | 1. Describe the characteristics of circulating COVID-19 variants.  2. Explain the purpose of COVID-19 vaccination.  3. Explain the effects of vaccination and prior infection on prevention.  4. Describe the epidemiology and clinical features of long COVID. | • COVID-19 epidemiological trends in 2022  • Characteristics of the Omicron variant (BA.5)  • Vaccination, reinfection, and breakthrough infections  • Long COVID | Online lecture, Q&A |
| Module 2 | Real-world clinical environment for respiratory disease care (30 min) | Instructor2 (professor of infectious diseases) | 1. Explain transmission routes of respiratory viral infections.  2. Identify environments vulnerable to airborne transmission.  3. Apply infection prevention strategies in clinical settings. | • Updated evidence on airborne transmission  • Ventilation and air purification strategies  • Infection control recommendations for respiratory diseases | Online lecture, Q&A |
| Module 3 | Emerging infectious diseases and the classification and reporting system for notifiable diseases (30 min) | Instructor3 (professor of infectious diseases) | 1. Understand emerging infectious diseases.  2. Identify transmission routes and PPE use for suspected mpox cases.  3. Recognize clinical manifestations of mpox.  4. Understand the classification system for notifiable infectious diseases.  5. Accurately report notifiable infectious diseases. | • Emerging and re-emerging infectious diseases  • Legal requirements for infectious disease reporting  • Class I and II notifiable infectious diseases  • Official reporting forms  • Clinical features and management of mpox | Online lecture, Q&A |
| Module 4 | Disinfection and sterilization of medical devices (30 min) | Instructor4 (professor of infectious diseases) | 1. Explain medical device reprocessing principles.  2. Select appropriate disinfection and sterilization methods.  3. Verify adequacy of reprocessing.  4. Apply reprocessing procedures in healthcare institutions. | • Medical device classification  • Levels of disinfection and sterilization  • Importance of cleaning and monitoring  • Storage and reprocessing management | Online lecture, Q&A |

COVID-19, coronavirus disease 2019.
