# Supplementary material for: Evaluation of an infectious‑disease response training program for primary care physicians in Korea using Kirkpatrick’s 4 levels and the Context, Input, Process, and Product model: a mixed‑methods study
Source: J Educ Eval Health Prof. 2025 Dec 31;22:40. doi: 10.3352/jeehp.2025.22.40 (PMC13006793; doi:10.3352/jeehp.2025.22.40)
Supplement: Supplementary file 3 — Supplement 2. Level 3 practical application (individual level: knowledge dissemination) at the 1-month follow-up: frequencies (n=100, %) and mean. [file jeehp-22-40-suppl2.docx]

**Supplement 2.** Level 3 practical application (individual level: knowledge dissemination) at 1-month follow-up: frequencies (n=100, %) and mean

| Item (explained/educated staff/patients on...) | Levels^a)^, % | | | | | Mean±SD |
| --- | --- | --- | --- | --- | --- | --- |
|  | 1 | 2 | 3 | 4 | 5 |  |
| 1. Characteristics of new COVID variants | 5 | 14 | 33 | 42 | 6 | 3.30±0.96 |
| 2. Main goals of COVID-19 vaccination | 4 | 17 | 31 | 40 | 8 | 3.31±0.98 |
| 3. Effect of vaccines/prior infection on prevention | 4 | 15 | 28 | 47 | 6 | 3.36±0.95 |
| 4. Patterns of Long-COVID | 4 | 17 | 31 | 43 | 5 | 3.28±0.94 |
| 5. Main transmission modes of respiratory viruses | 4 | 15 | 27 | 47 | 7 | 3.38±0.96 |
| 6. High-risk environments for aerosol spread | 5 | 15 | 28 | 46 | 6 | 3.33±0.97 |
| 7. Prevention of respiratory virus transmission in clinic | 4 | 13 | 33 | 42 | 8 | 3.37±0.95 |
| 8. Emergence and impact of novel diseases | 4 | 19 | 34 | 37 | 6 | 3.22±0.96 |
| 9. Mpox transmission & appropriate PPE | 11 | 26 | 34 | 23 | 6 | 2.87±1.08 |
| 10. Mpox clinical presentation & screening | 11 | 24 | 38 | 22 | 5 | 2.86±1.04 |
| 11. Reportable disease classification & reporting methods | 7 | 27 | 34 | 27 | 5 | 2.96±1.01 |
| 12. Concept and process of medical equipment reprocessing | 5 | 22 | 36 | 29 | 8 | 3.13±1.01 |
| 13. Appropriate disinfection/sterilization by equipment type | 5 | 22 | 36 | 28 | 9 | 3.14±1.03 |
| 14. Verifying sterilization status of reprocessed equipment | 5 | 23 | 34 | 28 | 10 | 3.15±1.05 |
| 15. Applying reprocessing procedures in the institution | 5 | 21 | 35 | 31 | 8 | 3.16±1.01 |
| Overall |  |  |  |  |  | 3.19±0.86 |

SD, standard deviation; COVID-19, coronavirus disease 2019; PPE, personal protective equipment.

^a)^Levels defined as 1=not at all; 2=rarely; 3=willing to explain/educate (motivation only); 4=mostly explained/educated; 5=very diligently explained/educated.
