# Supplementary material for: Evaluation of an infectious‑disease response training program for primary care physicians in Korea using Kirkpatrick’s 4 levels and the Context, Input, Process, and Product model: a mixed‑methods study
Source: J Educ Eval Health Prof. 2025 Dec 31;22:40. doi: 10.3352/jeehp.2025.22.40 (PMC13006793; doi:10.3352/jeehp.2025.22.40)
Supplement: Supplementary file 4 — Supplement 3. Level 3 practical application (individual-organizational level) at the 1-month follow-up (n=100). [file jeehp-22-40-suppl3.docx]

**Supplement 3.** Level 3 practical application (individual-organizational level) at 1-month follow-up (n=100)

| Item (response measures) | Levels^a),b)^ | | | | | Mean±SD |
| --- | --- | --- | --- | --- | --- | --- |
|  | 1 (%) | 2 (%) | 3 (%) | 4 (%) | 5 (%) |  |
| S1. Developed (COVID variants) | 7 | 10 | 52 | 27 | 4 | 3.11±0.90 |
| P1. Implemented (COVID variants) | 6 | 15 | 45 | 28 | 6 | 3.13±0.95 |
| S2. Developed (COVID vaccine goals) | 5 | 11 | 55 | 25 | 4 | 3.12±0.84 |
| P2. Implemented (COVID vaccine goals) | 4 | 15 | 50 | 26 | 5 | 3.13±0.87 |
| S3. Developed (vaccine/prior infection efficacy) | 4 | 14 | 44 | 31 | 7 | 3.23±0.92 |
| P3. Implemented (vaccine/prior infection efficacy) | 4 | 11 | 50 | 29 | 6 | 3.22±0.87 |
| S4. Developed (long-COVID patterns) | 5 | 12 | 54 | 22 | 7 | 3.14±0.90 |
| P4. Implemented (long-COVID patterns) | 4 | 16 | 51 | 22 | 7 | 3.12±0.90 |
| S5. Developed (respiratory virus transmission modes) | 4 | 11 | 47 | 31 | 7 | 3.26±0.89 |
| P5. Implemented (respiratory virus transmission modes) | 5 | 11 | 48 | 30 | 6 | 3.21±0.90 |
| S6. Developed (aerosol spread prevention) | 4 | 10 | 52 | 25 | 9 | 3.25±0.90 |
| P6. Implemented (aerosol spread prevention) | 4 | 12 | 48 | 29 | 7 | 3.23±0.90 |
| S7. Developed (clinic transmission prevention) | 5 | 10 | 51 | 27 | 7 | 3.21±0.90 |
| P7. Implemented (clinic transmission prevention) | 5 | 12 | 50 | 26 | 7 | 3.18±0.91 |
| S8. Developed (novel disease emergence) | 5 | 11 | 52 | 24 | 8 | 3.19±0.92 |
| P8. Implemented (novel disease emergence) | 5 | 11 | 54 | 22 | 8 | 3.17±0.91 |
| S9. Developed (mpox transmission & PPE) | 15 | 12 | 51 | 15 | 7 | 2.87±1.07 |
| P9. Implemented (mpox transmission & PPE) | 17 | 13 | 47 | 16 | 7 | 2.83±1.11 |
| S10. Developed (mpox clinical presentation) | 16 | 13 | 46 | 17 | 8 | 2.88±1.12 |
| P10. Implemented (mpox clinical presentation) | 19 | 12 | 46 | 16 | 7 | 2.80±1.14 |
| S11. Developed (reporting classification/methods) | 6 | 12 | 54 | 20 | 8 | 3.12±0.94 |
| P11. Implemented (reporting classification/methods) | 6 | 11 | 53 | 22 | 8 | 3.15±0.94 |
| S12. Developed (reprocessing concept/process) | 3 | 11 | 54 | 24 | 8 | 3.23±0.86 |
| P12. Implemented (reprocessing concept/process) | 5 | 11 | 50 | 25 | 9 | 3.22±0.94 |
| S13. Developed (disinfection/sterilization by type) | 4 | 13 | 46 | 29 | 8 | 3.24±0.92 |
| P13. Implemented (disinfection/sterilization by type) | 3 | 12 | 48 | 28 | 9 | 3.28±0.90 |
| S14. Developed (verifying sterilization status) | 3 | 9 | 51 | 27 | 10 | 3.32±0.89 |
| P14. Implemented (verifying sterilization status) | 5 | 9 | 49 | 27 | 10 | 3.28±0.94 |
| S15. Developed (applying reprocessing in clinic) | 5 | 11 | 50 | 25 | 9 | 3.22±0.94 |
| P15. Implemented (applying reprocessing in clinic) | 6 | 12 | 46 | 26 | 10 | 3.22±0.99 |
| Overall (S–development) |  |  |  |  |  | 3.13±0.83 |
| Overall (P–implementation) |  |  |  |  |  | 3.13±0.85 |

SD, standard deviation; COVID-19, coronavirus disease 2019; PPE, personal protective equipment.

^a)^S (strategy/development) level definitions: 1=no response measures developed; 2=1 or 2 measures developed; 3=basic measures developed; 4=relatively sufficient measures developed; 5=very sufficient measures developed. ^b)^P (practice/implementation) level definitions: 1=no measures implemented; 2=only partial implementation; 3=only basic implementation; 4=majority of measures implemented; 5=all measures implemented.
